# General surgeons’ attitudes towards COVID-19: A national survey during the SARS-CoV-2 virus outbreak

**DOI:** 10.1007/s10353-020-00649-w

**Published:** 2020-07-16

**Authors:** Justyna Rymarowicz, Tomasz Stefura, Piotr Major, Jacek Szeliga, Grzegorz Wallner, Michał Nowakowski, Michał Pędziwiatr

**Affiliations:** 1 2nd Department of General Surgery, Jagiellonian University Medical College, Jakubowskiego 2 st., 30-688 Krakow, Poland; 2 Department of General, Gastroenterological, and Oncological Surgery, Collegium Medicum Nicolaus Copernicus University, Torun, Poland; 3 2nd Department of General, Gastroenterological, and Oncological Surgery, Medical University, Lublin, Poland

**Keywords:** Personal protective equipment, Healthcare workers anxiety, SARS-CoV-2 virus, Surgical practice, Healthcare workers safety

## Abstract

**Background:**

The COVID-19 global pandemic left the unprepared health care systems struggling to mount a measured response. This gave rise to important questions about surgeons’ attitude towards surgical practice and the level of preparation at work.

**Methods:**

Cross-sectional web-based national survey distributed to general surgeons by e‑mail over a period of 7 days.

**Results:**

Among 304 responders, 42.6% were working in the hospital with COVID-19 patients. Three quarters of all surgeons (74.5%) were afraid of contracting the disease. While 42% expressed a fear for their own life while caring for COVID-19 patients, 90.1% were afraid of transmitting the disease to family members. The average reported level of PPE provided at the workplace was significantly higher among the group which was not afraid of contracting COVID-19 than among the group afraid of contracting COVID-19 (4.0 vs. 3.12, *p* = 0.02). Nearly all surgeons (93.8%) agreed that cancer surgeries should be continued during the pandemic and 49% perceived laparoscopy as a safe approach when operating on COVID-19 positive patients.

**Conclusion:**

A high proportion of surgeons admitted being afraid of working during the COVID-19 pandemic, which had various implications for their attitude towards surgical practice. Protecting health care workers is an important component of public health measures for addressing the epidemic

The COVID-19 pandemic has caused a major global health crisis. Health care systems internationally struggle to provide an adequate response to the current outbreak, while the number of people infected with a new coronavirus disease continues to grow exponentially [1]. Worldwide, a number of countries, Poland among them, transformed a portion of general hospitals into designated COVID centers, as one of the measures to contain disease spread and rationalize resources [2, 3]. Health care providers who work at the frontline are under the extreme pressure. Increased workload, fear for their families, scarcity of personal protective equipment, and a lack of access to rapid testing are among the main problems which may negatively influence their wellbeing at work [4]. Although the new coronavirus disease is not a surgical condition per se, given the number of infected individuals in the communities, a portion of patients with COVID-19 require surgical care. Surgical personnel are at an immense risk of exposure due to the high-risk procedures performed in the operating theatre [5]. Although multiple guidelines have been published by various international surgical societies regarding the appropriate management of surgical patients during the COVID-19 pandemic, they differ in various aspects [6–10]. The unprecedented situation may contribute to additional anxiety among surgical doctors, especially if they lack the required training and protective equipment. This study aimed to assess surgeons’ attitude towards surgical practice in relation to the hospitals’ preparations made during the early phase of the COVID-19 pandemic in the surgical wards of Polish hospitals.

## Materials and methods

We conducted an anonymous web-based national survey among Polish general surgeons from March 30 to April 6, 2020. The survey was conducted in collaboration with the Association of Polish Surgeons (APS) and the National Consultant in General Surgery and covered the domains of surgeons and their workplace characteristics, information sources, knowledge, risk perception, and preparedness related to the COVID-19 pandemic.

### Survey instrument

The 27-item self-administered questionnaire was designed based on the literature review of previous viral outbreaks [11, 12]. The questions were translated into Polish and adjusted to fit the COVID-19 outbreak and suit the local settings. Open-ended questions were limited to reduce information bias. The survey was sent to the president of the APS and the National Consultant in General Surgery to obtain their opinions regarding its simplicity and full content. This was followed by a pilot study, which was performed with 10 surgeons from our institution. According to that, the final questionnaire was determined by the authors.

The survey comprised four sections. The first captured demographic and workplace characteristics of the participants and whether they were exposed to the SARS-CoV‑2 virus. The second part assessed surgeons’ sources of information and asked responders to rate their level of knowledge about COVID-19 and the degree of adjustments made in their hospital. Respondents were asked to self-assess their level of general knowledge about COVID-19. The 1–10 rating scale was used, where 1 meant no knowledge about COVID-19 and 10 the knowledge at an expert level. Similarly, they were asked to evaluate the amount of COVID-19 pandemic-related training and personal protective equipment (PPE) provided at their workplace on the 1–10 rating scale, 1 being none provided and 10 above the required level.

The third section assessed responders’ perception of COVID risk. The fourth section asked the responders’ opinion about the surgery-specific queries such as changes in working hours, types of surgeries performed during the pandemic, and preferred surgical approach.

### Survey distribution

The survey was distributed via email to the APS members on March 30, 2020, and posted on two Polish online surgical discussion groups and the APS website.

### Inclusion and exclusion criteria

The study group included Polish general surgeons and surgical trainees working in public hospitals who granted informed consent to participate in the study. Retired surgeons, physicians, and trainees in surgical specializations other than general surgery, medical interns, medical students, and other health care professionals were excluded from this study.

### Data analysis

All data were analyzed using IBM SPSS Statistics (IBM Corp., Armonk, NY, USA). Descriptive statistics included frequency counts and percentages for categorical variables and means for continuous variables. Fear of contracting the disease and fear of death were dichotomized into yes vs. not/not sure. Quantitative data were compared made using Mann–Whitney’s test for non-parametric data and chi-square test for dichotomized outcomes. To determine independent variables which may correlate with the likelihood of fear of contracting COVID-19, a logistic regression was performed. All reported *p*-values are two tailed, and *p* < 0.05 was considered statistically significant.

## Results

Among 304 responders who were eligible and who fully completed the survey, the age ranged from 26–74 years (mean age 41.8 years), 30.6% were women. Of the responders, 41 surgeons (13.5%) reported being directly involved in the care of COVID-19 patients (18 surgeons working in COVID centers and 23 surgeons working in non-COVID centers), 130 (42.6%) were working in the hospital with COVID-19 patients (39 surgeons working in COVID centers and 90 surgeons working in non-COVID centers), and over a half of respondents (52.4%) declared having no COVID-19 patients in their hospital.

Professional literature, such as medical journals and WHO/ECDC guidelines, online discussion groups, and information at the workplace were the main self-reported sources of knowledge about COVID-19 disease (79.2%, 51.6%, and 49.3%, respectively; Table 1).

**Table 1:** Demographic workplace and source of knowledge characteristics. There were no missing data

Characteristics	*N*	(%)
*Age group (years)*		
Under 36	132	(43.4)
36–45	63	(20.7)
46–55	57	(18.6)
56+	52	(18.4)
*Gender*		
Male	211	(69.4)
Female	93	(30.6)
*Marital status*		
Married	220	(72.4)
Unmarried	84	(27.6)
*Children*		
Yes	197	(64.8)
No	107	(35.2)
*Career level*		
Specialist	194	(63.8)
Trainee	102	(33.6)
Other	8	(2.6)
*Hospital type*		
University	103	(33.9)
Regional	60	(19.7)
District	141	(46.4)
*Hospital category*		
COVID-19 center	52	(17.1)
Non-COVID-19 center	252	(82.9)
*Source of COVID-19 information (yes)*		
Professional literature (medical journals WHO/ECDC guidelines)	241	(79.3)
Online discussion groups	156	(51.3)
Workplace information	150	(49.3)
Social media	119	(39.1)
Television	115	(37.8)
Newspapers (printed/online)	92	(30.2)
Others	64	(21.0)

**Fig. 1:**
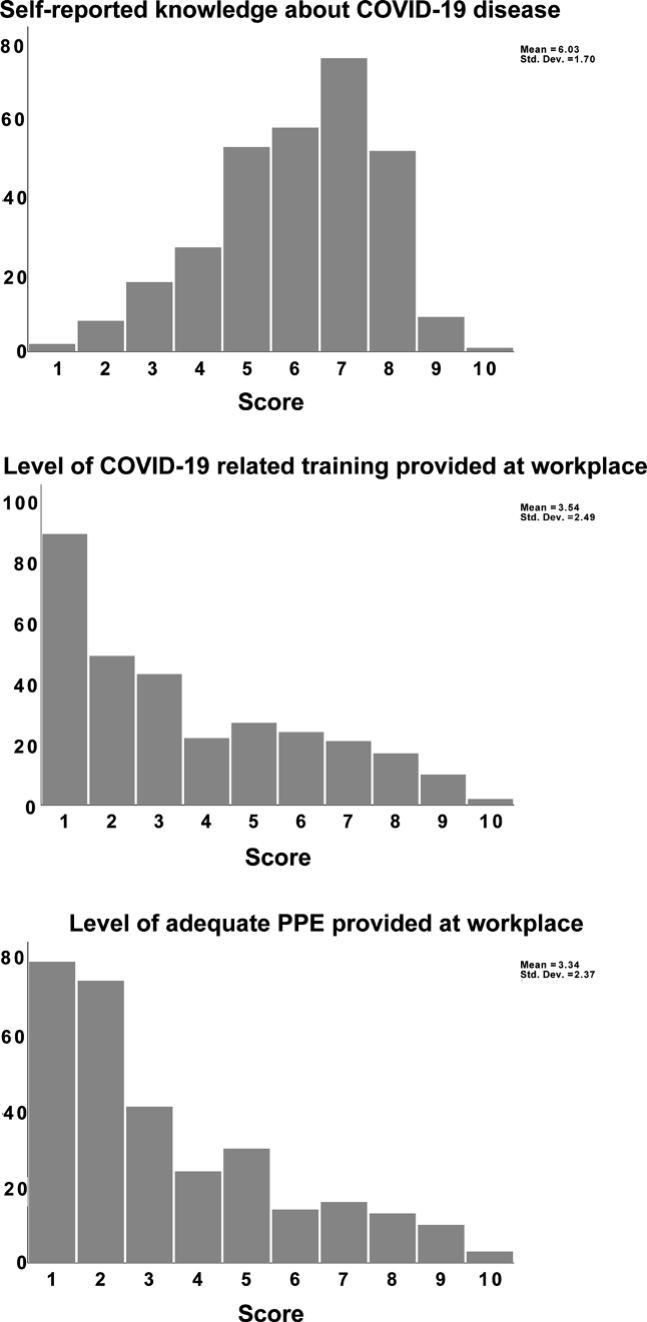
Self-reported level of knowledge and hospital preparation. The figure shows the number of surgeons who provided each response. There were no missing data

The mean self-reported knowledge about COVID-19 was 6.03 (SD = 1.7), with only 20.3% of surgeons assessing their knowledge at ≥8. The mean training provided at work during the new coronavirus outbreak was evaluated at 3.53 (SD = 2.49), with 59.5% of respondents marking it ≤3. Similarly, the mean mark for an adequate level of PPE provided at the workplace was 3.34 (SD 2.38), with 50.3% reporting it at ≤2, which means none or minimal PPE (Fig. 1).

**Fig. 2:**
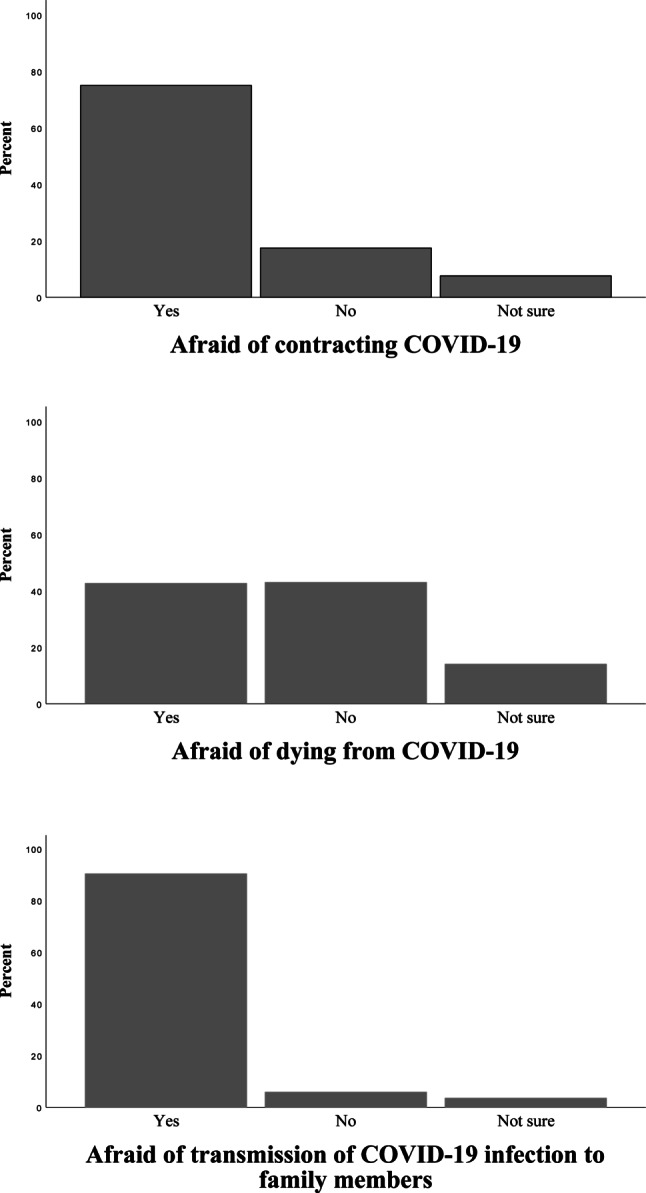
COVID-19 risk perception. The figure shows the percentage of surgeons who provided each response. There were no missing data

Two thirds (62.7%) of surgeons believed that they would contract COVID-19 during their work in the hospital. However, approximately three quarters of all (74.5%) were afraid of contracting the disease, mainly during the time of caring for in-hospital patients (184, 80.7%), from other staff members at work (184, 80.7%) and in out-patient clinics (87, 38.1%). Furthermore, 130 (42%) expressed a fear for their own life while taking care for COVID-19 patients. The great majority of respondents conceived that there is a high risk of transmitting SARS-CoV‑2 virus to family members (271, 89.1%) and they were afraid of transmitting the disease to the family members (274, 90.1%; Fig. 2).

The average age was significantly higher in the group who was afraid of dying from COVID-19 than in the group who was not afraid (43.33 vs. 40.79, *p* = 0.045). However, there were no statistically significant differences in age between surgeons who were afraid of contracting the disease compared to those who were not afraid (*p* = 0.98).

Similarly, the average reported level of PPE provided at the workplace was significantly higher in the group which was not afraid of contracting COVID-19 than among the group afraid of contracting COVID-19 (4.0 vs. 3.12, *p* = 0.02). Also, the average level of PPE provided at the workplace was significantly higher in the group which was not afraid of dying from COVID-19 compared to the group afraid of dying from COVID-19 (3.61 vs. 2.98 *p* = 0.016). Additionally, in the univariate regression model, the following factors were found to contribute to the fear of contracting the disease: age (odds ratio [OR] 0.99, 95% confidence interval [CI] 0.98–1.02), female sex (OR 1.11, 95% CI 0.63–1.97), having children (OR 1.02, 95% CI 0.61–1.70), specialist vs trainee (OR 1.11, 95% CI 0.63–1.97), university hospital vs. regional hospital vs. district hospital (OR 0.75, 95% CI 0.55–1.02), self-assessed level of knowledge about COVID-19 pandemic (OR 1.00, 95% CI 0.86–1.17), self-reported level of training provided at workplace (OR 0.96, 95% CI 0.87–1.07), adequate PPE supply at workplace (OR 0.86, 95% CI 0.78–0.96%). These factors were used to build a multivariable regression model which revealed that solely an adequate PPE supply at the workplace was an independent variable influencing the fear of contracting COVID-19.

There were no statistically significant associations between the fear of contracting the disease or dying from COVID-19 and gender, career level, the level of knowledge about COVID-19, and the amount of COVID-19 training provided at the workplace. Likewise, it was irrelevant whether respondents were working in COVID centers or had been directly involved in the care of patients suffering from COVID-19 or not.

A total of 182 surgeons (59.9%) reported having an additional employment, such as private practice, outpatient clinics, or on-call shifts in different hospitals. Among them, 32% limited and 53.2% temporarily discontinued their additional employment. Similarly, when asking about the rota changes, 83.3% of respondents thought that the surgical rota should be switched to an on-call rota only and 41% believed that surgeons over 60 years old should be exempted from work during the pandemic, with only 10% believing that the normal rota shifts should be continued.

Similarly, only a minor proportion of surgeons (11.1%) were likely to continue all planned surgeries for patients with a confirmed COVID-negative RT-PCR swab test. However, nearly all (93.8%) agreed that cancer surgeries should be continued, either for all cancer patients (36.9%) or for those with confirmed COVID-negative RT-PCR swab tests (56.2%). When asking about the preferred surgical technique in conditions where the laparoscopic approach has been well established as a gold standard, such as cholecystectomy or appendicectomy, 149 respondents (49%) selected laparoscopy as the method of choice when performing this type of surgery on COVID-positive patients. While 89 surgeons (29.3%) would, in this situation, use the open technique and 66 surgeons (21.8%) either did not know which approach is safer or believed that there is no difference.

## Discussion

To our knowledge, this is the first study that aimed to assess surgeons’ attitudes towards surgical practice in relation to hospitals’ preparations made during the early phase of the COVID-19 pandemic in the surgical wards in Polish hospitals. Appropriate level of PPE and age were important factors influencing whether surgeons were more likely to be afraid of COVID-19. The great majority of surgeons believed that the surgical rota should be adjusted to minimize their risk of exposure.

While professional literature was the most frequently used source of information, only half of responders were provided with information about the new coronavirus pandemic at their workplace. Interestingly, online discussion groups were the second most common source of information about COVID-19, and social media were more popular than television and newspapers. Using an online source as a main reference point has the undoubted advantage of being able to keep up to date, which is particularly important during such a dynamic situation as the COVID-19 pandemic. However, they may not be as well verified as printed information and therefore may lack credibility.

Furthermore, our study revealed that an overwhelming proportion of surgeons reported fear of contracting COVID-19 along with a significant number of respondents expressing a fear for their own life while working during the pandemic. Surgeons who were afraid of death were older than those who were not afraid. This may be explained by the fact that advanced age strongly correlates with the likelihood of suffering a more severe form of COVID-19 and that the case fatality rate is significantly higher among people over 65 years old [13, 14]. Perhaps this is why nearly half of respondents whished that surgeons over 60 years of age were exempt from work during the pandemic.

The amount of PPE provided to surgical wards stood at a staggeringly low level, as by the time the epidemic started in Poland, the global stock of PPE was already depleted and the country struggled to acquire an adequate number of masks and gowns. According to the national strategy implemented by the Ministry of Health in Poland, the treatment of all patients with confirmed COVID-19 should occur in designated COVID centers; therefore, those institutions were prioritized when supplying hospitals with PPE. The remaining hospitals received a limited supply of PPE. Moreover, an adequate level of PPE was the only contributing factor which was significantly associated with the fear of contracting the disease among surgeons. Our findings are in keeping with a recently published letter from one of the hospitals in China, where authors indicated that adequate PPE and uninterrupted rest were the only two requirements indicated by healthcare providers working during the outbreak [15]. Similarly, Shanafel et al. placed access to appropriate personal protective equipment as one of the main concerns among frontline healthcare providers [4]. Therefore, sufficient stock of PPE in every hospital should become a priority if we wish to minimize the level of fear among surgeons working during the pandemic and the possible long-term consequences of working in an unsafe environment [16].

Interestingly, at the time of this study, a significant proportion of patients suffering from COVID-19 were still hospitalized in non-COVID-dedicated hospitals. This may explain why surgeons working in the hospitals which are not assigned for treatment of COVID-19 patients displayed the same level of anxiety as surgeons working in dedicated COVID centers.

The great majority of surgeons preferred replacing normal rota hours with the on-call rota only during the peak of the pandemic, which may also indirectly indicate their unwillingness to attend work during the outbreak. Similarly, a substantial percentage believed that only lifesaving procedures, such as emergency and cancer surgeries, should be continued in this period.

Finally, the survey revealed differences in the perceived safety of laparoscopy. This may reflect the fact that the guidelines published by various international surgical bodies at the time of our survey were inconsistent in this matter. While the European Association of Endoscopic Surgery considered laparoscopy safe for SARS-CoV-2-infected patients, the Royal College of Surgeons in England advised an open technique for every procedure performed on COVID-19 patients [6, 10]. The evidence against laparoscopy was relatively weak and derived from research performed on different pathogens [17, 18]. Still, a high proportion of surgeons preferred an open approach, which may support the theory that their clinical decision was driven more by the fear for their personal safety than it was based on well-established evidence.

### Limitations

The study has several limitations. First, while the study population was representative of membership in the nation’s largest surgical organizations, it may not represent all Polish surgeons. Second, the study was conducted in an early phase of the pandemic and was carried out during 7 days; therefore, the situation may be a subject to change. Third, the survey used an unvalidated questionnaire and the anxiety level was not established based on psychometric tests; therefore, about its long-term consequences are of limited value. This can represent, however, a pilot study for future research. Fourth, a major limitation was also a self-assessment of knowledge and subjective opinions about workplace adaptations which may therefore be prone to bias. Finally, we were unable to calculate the response rate of the participants as the survey was not only sent to individual email addresses, but also published on the surgical websites; therefore, we cannot estimate how many surgeons could have participated in it.

## Conclusion

A high proportion of surgeons admitted being afraid of working during the COVID-19 pandemic, which had various implications for their attitude towards surgical practice. Protecting health care workers is an important component of public health measures for addressing the epidemic. Lack of adequate support may result in serious mental health consequences and surgeons’ unwillingness to continue their practice.
